# Bladder Smooth Muscle Cells Differentiation from Dental Pulp Stem Cells: Future Potential for Bladder Tissue Engineering

**DOI:** 10.1155/2016/6979368

**Published:** 2016-01-06

**Authors:** Bing Song, Wenkai Jiang, Amr Alraies, Qian Liu, Vijay Gudla, Julia Oni, Xiaoqing Wei, Alastair Sloan, Longxing Ni, Meena Agarwal

**Affiliations:** ^1^School of Dentistry, Cardiff Institute of Tissue Engineering and Repair, Cardiff University, Heath Park, Cardiff CF14 4XY, UK; ^2^State Key Laboratory of Military Stomatology, Department of Operative Dentistry & Endodontics, School of Stomatology, Fourth Military Medical University, Shaanxi 710032, China; ^3^Department of Urology/Surgery, Cardiff University and University Hospital of Wales, Heath Park, Cardiff CF14 4XW, UK

## Abstract

Dental pulp stem cells (DPSCs) are multipotent cells capable of differentiating into multiple cell lines, thus providing an alternative source of cell for tissue engineering. Smooth muscle cell (SMC) regeneration is a crucial step in tissue engineering of the urinary bladder. It is known that DPSCs have the potential to differentiate into a smooth muscle phenotype in vitro with differentiation agents. However, most of these studies are focused on the vascular SMCs. The optimal approaches to induce human DPSCs to differentiate into bladder SMCs are still under investigation. We demonstrate in this study the ability of human DPSCs to differentiate into bladder SMCs in a growth environment containing bladder SMCs-conditioned medium with the addition of the transforming growth factor beta 1 (TGF-*β*1). After 14 days of exposure to this medium, the gene and protein expression of SMC-specific marker (*α*-SMA, desmin, and calponin) increased over time. In particular, myosin was present in differentiated cells after 11 days of induction, which indicated that the cells differentiated into the mature SMCs. These data suggested that human DPSCs could be used as an alternative and less invasive source of stem cells for smooth muscle regeneration, a technology that has applications for bladder tissue engineering.

## 1. Introduction

Bladder augmentation or replacement is required in a variety of urological disorders including cancer, spinal injury, and benign bladder contracture. Hitherto, there is no suitable substitute available to restore the normal function of Detrusor muscle of native bladder, which allows expansion for storage of urine under low pressure (compliance) and contraction for voiding. Augmentation cystoplasty to increase the bladder capacity or substitution cystoplasty to replace the bladder is currently performed using a piece of reconfigured bowel, which has associated morbidity due to loss of contraction, thus necessitating intermittent self-catheterization to empty the bladder. There is mucous production and absorption of electrolytes by the bowel mucosa, in addition to causing infections, stones, and posing a small risk of cancer at the bowel/bladder interface, thus making it far from an ideal substitute. The ability to regenerate urological tissue with functions similar to the native bladder tissue would be a step forward in reducing the morbidity associated with the use of bowel, thus providing a better quality of life for the patients.

Attempts have been made to construct an autologous engineered bladder by obtaining urothelial and smooth muscle cells from bladder biopsy, growing the cells in* in vitro* cultures, and then seeding them onto a biodegradable bladder-shaped scaffold before implanting in the patients [1]. However, functionally these reconstructed bladders do not behave in the same way as the native bladder.

Although bladder smooth muscle cells (SMCs) are easy to obtain by bladder biopsy, they do not show any useful expansion* in vitro* [2, 3]; furthermore the cells obtained from older individuals have a reduced capability for replication [3]. Therefore alternative cell sources are required for any meaningful regeneration of urinary bladder tissue. Dental pulp stem cells (DPSCs) are mesenchymal-derived stem cells arising from the perivascular niche of dental pulp. Compared with other mesenchymal stem cells (MSCs) derived from bone marrow, adipose tissue, peripheral blood, and umbilical cord blood, DPSCs have marked advantage due to its accessibility with least invasive procedures without any ethical issues. Previous studies proved that DPSCs have potential for odontogenic [4], endothelial [5], myogenic [6, 7], hepatocytic [8], and melanocytic differentiation [9]. Due to this potential versatility, DPSCs are considered to be multipotent stem cells that can be used for a variety of therapeutic applications.

Previous studies has demonstrated that several lines of undifferentiated mesenchymal stem cells (MSCs), such as MSCs derived from adipose tissue [10] and bone marrow [11], have already expressed the SMC markers such as alpha smooth muscle actin (*α*-SMA), desmin, and h1-calponin. Belonging to one of MSCs, DPSCs could be another useful precursor for SMC differentiation as they also share certain SMC markers, for example, *α*-SMA. This has been demonstrated to be upregulated when damage occurs to the teeth, thus allowing regeneration [12]. Previous studies have shown that there are various clones of DPSCs, some losing their regenerative abilities faster than others. In this study, we isolated and selected one of the human DPSCs clones, which are able to maintain high proliferation abilities over a long period of time, making them beneficial for the future tissue engineering. Furthermore, bladder tissue engineering so far has utilised a variety of stem cells including those from embryo [13, 14], bone marrow [15], and adipose tissue [10]. These stem cells have been differentiated into the urothelial cells and smooth muscle cells, which make up the layers of the urinary bladder. However, these cell sources are associated with ethical problems, invasive procedure, or inadequate expansion capability. The usage of human DPSCs has the advantage of easy ethics approval and therefore could potentially benefit more in the future application in regenerative medicine.

It has been demonstrated that DPSCs have the potential to differentiate into a smooth muscle phenotype* in vitro* using differentiation agents, and most of these studies are focused on the vascular SMCs. However it is unclear whether human DPSCs could be differentiated into bladder specific SMCs in order to accommodate the urological functions in the future regeneration medicine application. It is known that cells release cytokines to neighbouring tissues, which can modulate the fate of adjacent cells via paracrine signaling. A coculturing method therefore is usually used* in vitro* to induce stem cells to differentiate toward fully functional cells when the induced growth factors are unknown. This study aims to examine whether stem cells arising from the human dental pulp can be induced to differentiate into bladder SMCs by using the conditioned medium (CM) from bladder SMCs and therefore have a potential clinical application in the tissue engineering of urinary bladder. We present a reliable and reproducible method of DPSCs differentiation into SMCs.

## 2. Materials and Methods

### 2.1. The Isolation of Human DPSCs Clone A32 and Human SMCs

The clonal populations of DPSCs were isolated using fibronectin based selection protocol as described previously [16, 17]. Briefly, the pulp tissues were obtained from third molars (donors aged from 17~20 years) with the patient's informed consent and ethical approval by the South East Wales Research Ethics Committee of the National Research Ethics Service (permission number: 07/WESE04/84). Then the pulp tissues were digested in a 4 mg/mL solution of collagenase/dispase (Roche Diagnostics) for 1 hour at 37°C. Following centrifugation and resuspension in alpha modification of Eagle's medium (*α*-MEM) supplemented with 100 units/mL penicillin, 100 *μ*g/mL streptomycin, 20%  (v/v) heat-inactivated foetal bovine serum (FBS) (all Life Technologies), and 100 *μ*M l-ascorbic acid 2-phosphate (Sigma-Aldrich), a single cell suspension was obtained by passing through a 70 *μ*m pore mesh cell strainer (BD Biosciences). Cells were seeded at 4000 cells/cm^2^ onto 6-well culture plates, previously coated with 10 *μ*g/mL fibronectin (Sigma-Aldrich), and incubated at 37°C in 5% CO_2_ for 20 minutes. The clones of cells that adhered to fibronectin were selected. Following 12 days of culture, with medium changes every 2-3 days, individual colonies detached using cloning rings and accutase (Life Technologies). Single cell-derived clones were expanded in culture by seeding at a density of 4000 viable cells/cm^2^ on culture dishes of increasing surface area. The level of population doublings (PD) during expansion culture was monitored and the clone which could proliferate steadily for up to 300 days of culture reaching 80+ PD was selected, named A32:

(1) $$\begin{array}{c} \text{PD}=\frac{\log_{10}\left(\text{total cell count obtained}\right)-\log_{10}\left(\text{total cell count re-seeded}\right)}{\log_{10}\left(2\right)}. \end{array}$$

Human SMCs were obtained from the bladder of patients who underwent open procedures for their bladder, after patient consent and ethical approval by the South East Wales Research Ethics Committee of the National Research Ethics Service (permission number: 07/WESE04/84). The human bladder tissue obtained was repeatedly washed with phosphate buffered saline (PBS) supplemented with 1% penicillin/streptomycin. Following this the bladder tissue was incubated overnight on a petri dish in 1000 U/mL dispase 2 (Roche) at 4°C. After 12 hours the bladder tissue was again washed repeatedly with PBS and the smooth muscle layer separated from urothelial layer. The bladder muscle was minced into 1 × 1 mm pieces and digested in collagenase type IV enzyme 250 IU/mL (Sigma-Aldrich) for 30 minutes at 37°C. The cell suspension was sieved through a 70 *μ*m filter and suspended in Dulbecco's modified Eagle's medium (DMEM), penicillin/streptomycin, and 10% FBS. The SMCs were seeded in a flask at a density of 3000 cells/cm^2^. Thereafter the SMCs were incubated at 37°C in 5% CO_2_. After 24 hours the medium was changed and thereafter the medium was changed every 2-3 days.

### 2.2. Differentiation of Human A32 DPSCs

Differentiation of the A32 was induced by using CM collected from bladder SMCs, supplemented with transforming growth factor beta 1 (TGF-*β*1), similarly as previously described [15, 18, 19]. CM was obtained by culturing SMCs in DMEM and 10% FBS until they reached passage 8. When they reached 70% confluence the medium was changed to *α*-MEM and 15% FBS. After 48 hours the medium was removed, centrifuged at 1500 rpm for 5 minutes, and sieved through a 40 *μ*L filter. Initially various concentrations of CM were used to ascertain which were the most potent inducers of differentiation. Concentrations of 0%, 10%, 20%, and 50% were trialled. It was found that a concentration of 20% and 50% CM induced the most differentiation of DPSCs to SMCs. Therefore 20% CM was chosen as this required less passaging of SMCs to retrieve the media. Many papers have used a protocol of addition of growth factors (e.g., TGF-*β*1) to media in order to induce stem cells to differentiate into SMCs [15, 18, 19]. We chose TGF-*β*1 at 2.5 ng/mL in DMEM and 15% FBS for the SMC differentiation protocol based on our pilot titration experiment. Previous studies also documented that addition of L-ascorbic acid at 30 *μ*M works symbiotically with TGF-*β*1 [18]. However this did not appear to be the case in our preliminary studies and therefore was excluded from the protocol. Therefore it was decided to use 2.5 ng/mL TGF-*β*1 and 20% CM as an optimised SMCs differentiation protocol.

DPSCs were cultured with a 1 : 1 medium mixture containing DMEM/15% FBS/20% CM, with the other DMEM medium (15% FBS, 2.5 ng/mL TGF-*β*1). Following the indicated time (0, 5, 8, 11, and 14 days) of incubation, the morphology, mRNA, and protein levels were evaluated compared to SMCs and noninduced DPSCs, grown in their optimised medium to assess their differentiation.

### 2.3. Immunocytochemistry

Cells were fixed with 4% PFA for 30 min and then incubated in PBS containing 0.4% Triton X-100 for 10 min on ice and then blocked with bovine serum albumin (BSA) for 60 min at 37°C. After the blocking step, the cells were incubated with primary antibody, anti-vimentin (1 : 100), anti-keratin (1 : 100), anti-STRO-1 (1 : 100), anti-myosin (1 : 50), anti-alpha-SMA (1 : 100), and anti-desmin (1 : 50) at 4°C overnight; PBS was used as the negative control. The cells were then washed with PBS and incubated for 1 h with the secondary antibodies, namely, anti-mouse IgG Alexa Fluor-488 or at anti-rabbit IgG Alexa Fluor-594 1 : 1000 at room temperature. Glass cover slips were mounted using mounting media supplemented with DAPI stain (VectorLabs) and preparations imaged under a fluorescent microscope.

### 2.4. Reverse Transcriptase PCR (RT-PCR) and Real Time Quantitative PCR (qPCR)

Total RNA was extracted from the cells using an RNeasy Mini Kit (QIAGEN) according to manufacturer's directions. The total yield of RNA per extraction was calculated using a Nanovue spectrophotometer (GE Healthcare) to measure the absorbance at 260 nm. A260/A280 ratios of 1.9–2.1 indicated extraction of good quality RNA. CDNA was synthesised from 2000 ng RNA using MMLV reverse transcriptase (Promega). PCR reactions were performed on DPSCs using GoTaq Polymerase (Promega) and the product specific primers CD105, CD73, CD44, CD90, and CD34 listed in Table 1 under the following cycling conditions: 1 minutes denaturation at 95°C followed by 1 minutes annealing at 60°C and 1.5 minutes elongation at 72°C for 30 cycles. The housekeeping gene D-glyceraldehyde-3-phosphate dehydrogenase (GAPDH) was used as positive control. PCR products were visualized under UV light following electrophoresing in 1.4% (w/v) agarose/TAE gel.

For qPCR readings, three separate cDNA samples were used and each measured in triplicate. Target-specific primers (Table 1) were added to each cDNA sample together with Precision MasterMix with ROX and SYBRgreen (PrimerDesign). Readings were taken using an ABI Prism fast 7500 qPCR machine (Advanced Biosystems) under the following cycling conditions: an initial denaturation step of 95°C for 2 minutes followed by 40 cycles of 15 seconds denaturation (95°C) and 1 minute annealing/elongation at 60°C. The relative amount or fold change of the target gene expression was normalized relative to the level of GAPDH and relative to a control (non-induced cells).

### 2.5. Western Blot Analysis

The total protein content was extracted from the cells by using lysis buffer containing protease inhibitors (Sigma-Aldrich, USA). The protein concentration was measured by using a BCA-200 protein assay kit (Pierce, Rockford Ill., USA). Equal amounts of protein were separated by sodium dodecyl sulfate/polyacrylamide gel electrophoresis and transferred to a polyvinylidene fluoride membrane (PVDF). The membrane was blocked in TRIS-buffered saline with Tween (TBST) containing 5% nonfat dry milk for 2 h and probed with primary antibodies myosin (1 : 500, Sigma), *α*-SMA (1 : 500, Sigma), desmin (1 : 500, Sigma), and GAPDH (1 : 1000, Sigma) overnight at 4°C and then incubated for 2 h with a horseradish-peroxidase-conjugated anti-mouse IgG antibody or anti-rabbit IgG diluted 1 : 20,00 (Sigma). Protein bands were visualized on X-ray film by using an enhance chemiluminescence system (GE Healthcare, Buckinghamshire, UK). The relative protein expression intensities were quantified by densitometry by using Quantity One analysis software.

### 2.6. Statistical Analysis

Each experiment was performed at least three times, unless otherwise indicated. Data are reported as the mean ± SE (standard error) deviation from three independent experiments. The significance of the differences between the experimental and the control groups was determined by using one-way analysis of variance; *P* < 0.05 indicated statistical significance.

## 3. Results

### 3.1. Isolation and Characterization of DPSC Clone A32

**Figure 1:**
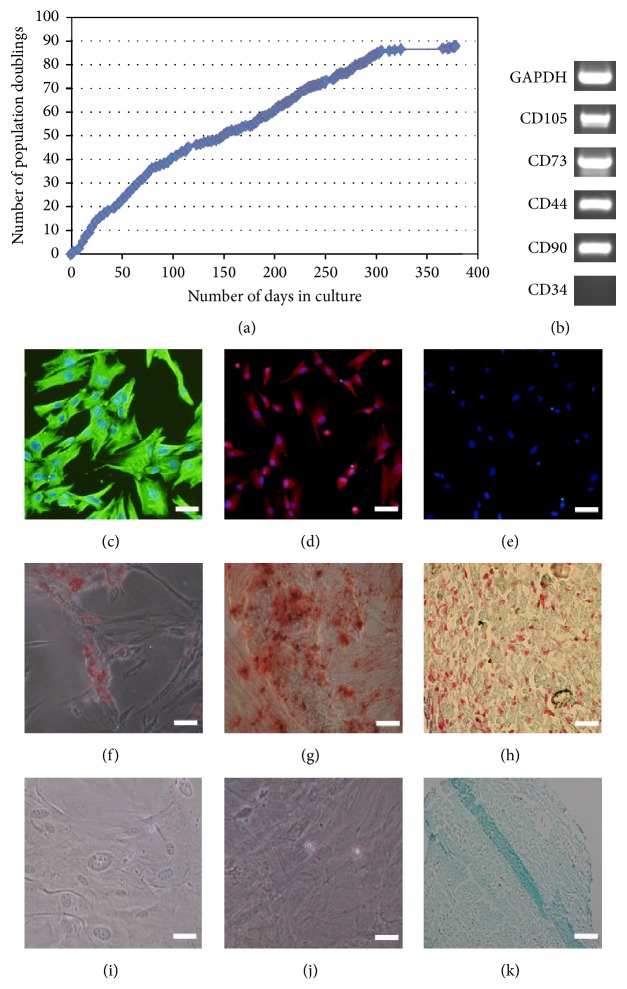
Characterization of human dental pulp stem cells (DPSCs) clone A32. The population doublings of DPSCs clone A32 exceed 80+ over 300 days in culture (a). The characterization of A32 by Rt-PCR and immunocytochemical staining: positive for the markers of CD105, CD73, CD44, and CD90 (b), negative for the marker of CD34 (b), positive immunostaining for vimentin (c) and STRO-1 (d), and negative immunostaining for keratin (e). This clone was able to differentiate into the 3 mesenchymal lineages: adipogenic (oil-red o staining) (f), osteogenic (alizarin red staining) (g), and chondrogenic (safranin o staining) (h) when cultured in appropriate differentiation conditions* in vitro* compared to control groups ((i)–(k)), respectively.

Dental pulp cells were successfully isolated from pulp tissue of extracted third molars. The clones of cells that adhered to fibronectin were selected. Single cell-derived clones were expanded in culture by seeding at a density of 4000 viable cells/cm^2^ on culture dishes of increasing surface area. The clone, which could proliferate steadily for up to 300 days of culture reaching 80+ PD, was selected, named A32 (Figure 1(a)). Then A32 was characterized by Rt-PCR, immunocytochemical staining, and multiple lineage differentiation tests. The result of Rt-PCR showed that A32 were found to express a range of mesenchymal stem cell markers including CD105, CD73, CD44, and CD90 but not to express the marker of CD34 (Figure 1(b)). Immunocytochemical staining of A32 revealed that the cells positively expressed vimentin (Figure 1(c)) and STRO-1 (Figure 1(d)) and were negative for keratin expression (Figure 1(e)). The multiple lineage differentiation tests revealed that A32 stained positive for lipid droplets with oil-red o after 5 weeks of adipogenic induction (Figure 1(f)). After 3 weeks of osteogenic induction and chondrogenic induction, A32 stained positive for mineral nodules with alizarin red S (Figure 1(g)) and chondrogenic with safranin o S (Figure 1(h)).

### 3.2. Change in Cell Morphology after SMC-Induction

**Figure 2:**
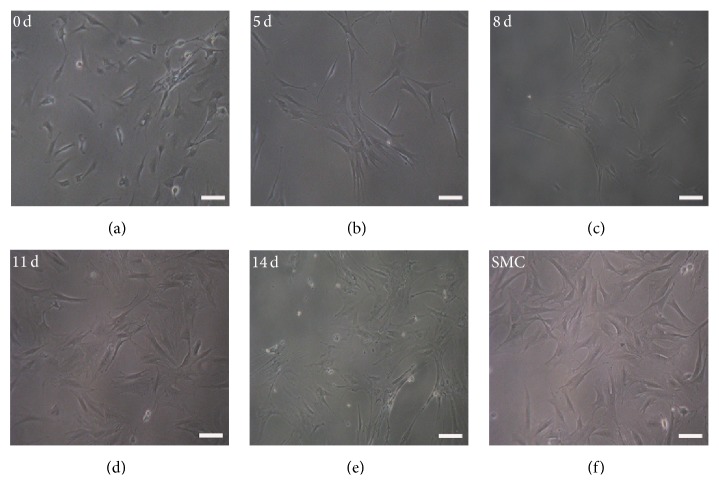
Morphology changes of human dental pulp stem cell (DPSC) clone A32 following an SMC-induction protocol. The morphology is demonstrated to change from the spindle shape of the A32 to the “hill and valley” morphology of SMCs over time ((a)–(e)). The SMC as the positive control (f). The noninduced A32 as the negative control (a). Bar 50 *μ*m.

The A32 were cultured in the differentiation medium for 14 days. The morphology of A32 was changing according to the time course (Figures 2(a)–2(e)). After 11 days, most of the cells began to display the typical “hill and valley” (Figure 2(d)) compared with the morphology of SMCs (Figure 2(f)). Cells in the control group maintained the spindle shape characteristic to DPSCs (Figure 2(a)).

### 3.3. Expression of Main SMCs Markers Using Immunocytochemistry

**Figure 3:**
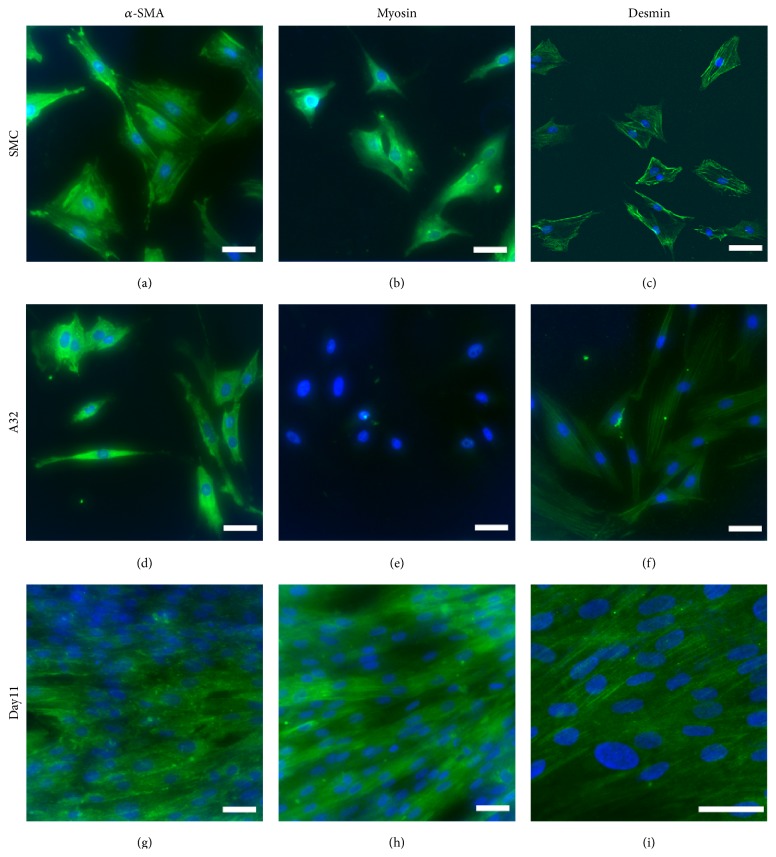
Immunocytochemistry of main SMCs markers in human dental pulp stem cell (DPSC) clone A32 after SMC-induction. The induced cells were stained for the SMCs markers of *α*-SMA, myosin, and desmin for 11 d ((g)–(i)). Noninduced A32 as the negative control ((d)–(f)). The SMCs as the positive control ((a)–(c)). The green staining indicates a positive result. Nuclei were stained with DAPI. Bar 50 *μ*m.

Noninduced A32 were found to express *α*-SMA (Figure 3(d)) and desmin (Figure 3(f)) already, but none of these cells stained positive for myosin (Figure 3(e)). The expression of *α*-SMA and desmin appeared to increase with the cells cultured in the differentiation medium over time. The pictures of 11 days differentiation are shown (Figures 3(g) and 3(i)). The differentiated cells stained positive for myosin after 11 days of induction (Figure 3(h)). The SMCs staining positive for *α*-SMA, myosin, and desmin were regarded as the positive control (Figures 3(a)–3(c)).

### 3.4. The Expression of SMC-Specific Markers in Differentiated A32

**Figure 4:**
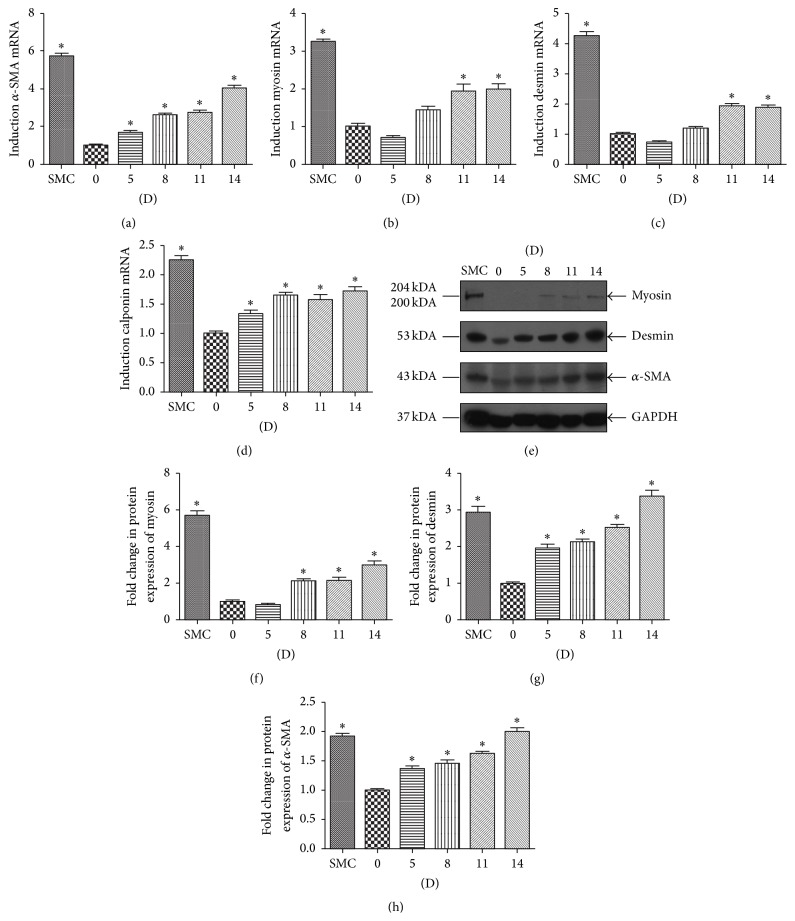
The expression of SMCs markers in human dental pulp stem cell (DPSC) clone A32 after SMC-induction. The A32 were induced in the SMCs induction protocol of 20% conditioned medium and 2.5 ng/mL TGF-*β*1 for the indicated time (0 d, 5 d, 8 d, 11 d, and 14 d). SMCs were regarded as a positive group. Noninduced A32 was regards as negative control. The mRNA expression of *α*-SMA, myosin, desmin, and calponin was analysed by qRT-PCR ((a)–(d)) and the protein levels of *α*-SMA, myosin, desmin were analysed by western blotting (e). The relative band intensities were determined by densitometry ((f)–(h)). Statistical analysis was performed by using one-way ANOVA. Date are shown as means ± SE. ^*∗*^ *P* < 0.05 when compared with the 0 D group.

The time course (0 d, 5 d, 8 d, 11 d, and 14 d) to detect myosin, *α*-SMA, desmin, and calponin mRNA expression in A32 in response to the induction of the differentiation medium was performed. The mRNA expression of *α*-SMA (Figure 4(a)) and calponin (Figure 4(d)) appeared to increase over time. The mRNA expression of myosin (Figure 4(b)) and desmin (Figure 4(c)) increased after 11 days of differentiation and maintained their maximal induction until 14 days. The mRNA expression of SMCs was regarded as the positive control. The protein level of myosin, *α*-SMA, and desmin was analysed by western blotting. The protein level of myosin began to increase after 8 days of induction and reached its maximal induction at 14 days (Figures 4(e) and 4(f)). The protein level of desmin (Figures 4(e) and 4(g)) and *α*-SMA (Figures 4(e) and 4(h)) appears to increase over time after SMC-induction. They reached their highest level after 14 days of induction. The protein expression of SMCs was regarded as the positive control.

## 4. Discussion

The urinary bladder wall is composed of Detrusor smooth muscle arranged in three distinct layers responsible for its compliance to store urine under low pressure and contraction for voiding, lined by a layer of transitional cells that provide a barrier to absorption. For many patients requiring augmentation or removal of the bladder, a piece of bowel is used. This however does not provide the same function as the native bladder, and there is much associated morbidity. Tissue engineering to generate the bladder wall components with properties similar to native tissues will provide a solution to this problem. Many advances have been made in tissue engineering of the urinary bladder using stem cell technology. Previous studies obtained autologous SMCs directly from the bladder muscle biopsy. However, it poses many experimental challenges, apart from the clinical need of invasive procedures to obtain a biopsy. It is extremely difficult to establish a sufficiently healthy cell population when obtained by biopsies from patients with end-stage bladder disease or from older individuals who are the most likely candidates requiring such bladder replacement or augmentation [3].

Additionally, patients with bladder tumours cannot use their own SMCs, due to the high potential of tumour cells contamination. Numerous investigators have tried to find alternative source of stem cells that can be induced to differentiate into the SMCs to replace the autologous SMCs. Differentiation protocols using cells from adipocytes [10], embryonic cells [13, 14], bone marrow [15], and urine [19] have been described for bladder tissue engineering experiments. Hitherto, none of the studies have assessed the potential of human DPSCs differentiating into bladder SMCs. Compared with the above-mentioned sources of cells, DPSCs demonstrated advantages of easy access with least invasive procedures, without ethical issues, high proliferation, excellent regeneration, and multiple-potential of differentiation as well as little inherent immunogenicity, establishing DPSCs as a promising cell source for the tissue engineering and regenerative medicine experiments. The differentiation potential of DPSCs is not limited to mesenchymal cell type only; there is evidence of differentiation potential for endothelial [5], myogenic [6, 7], hepatocytic [8], and melanocytic routes [9]. Therefore, the potential clinical application of DPSCs is not only in regenerative dentistry, but also in regenerative treatments for other systems [20], such as neuroplasticity for central nerve disease [21–23] and spinal cord injury [24], myogenic regeneration for muscular dystrophy and myocardial infractions [5, 25], and osteogenic regeneration for calvarial bone [26, 27], mandibular bone [28, 29], and alveolar bone [30]. Additionally, DPSCs have been proved to be tolerated as an allogeneic cell transplant without the need for immunosuppression, consequently bypassing the issue of patient-matched autologous applications [31]. The clone of A32 that we isolated from a number of clones of human DPSCs is particularly versatile. It not only has potential to differentiate into all three mesenchymal lineages but also expressed the SMC-related markers including *α*-SMA and desmin, indicating its potential to differentiate into SMCs. One of the most important advantages of A32 is its high proliferation capacity for up to 300 days of culture (80+ PD), which makes it more valuable for tissue engineering. We demonstrated in this study that the DPSCs clone A32 can be induced to differentiate into bladder SMCs using a human bladder SMCs-CM model in combination with TGF-*β*1, demonstrated by the “hill and valley” morphology change after 11 days of induction, and confirmed by *α*-SMA, desmin, myosin, and calponin expression. We further found that *α*-SMA was already present in noninduced DPSCs, which is similar to findings in human bone marrow mesenchymal stem cells (BMMSCs) and human adipose-derived stem cells [15], but not in human embryonic stem cells [13]. It was upregulated when the cells were differentiated. *α*-SMA is an early marker of developing smooth muscle, which does not provide definitive evidence for a smooth muscle lineage. Thus, to further characterize myogenic differentiation thoroughly, we chose to evaluate not only *α*-SMA but also other smooth muscle markers, particularly myosin which is not detected in any other cell type and is only expressed in contractile SMCs [32, 33]. The expression of myosin was not seen in the noninduced DPSCs and early stage of induction but was present after 11 days of differentiation, indicating the differentiation into the mature SMCs. Desmin is a muscle-specific intermediate filament that plays an important role in integrating the sarcolemma, Z disk, and nuclear membrane in sarcomeres and regulating sarcomere architecture [34]. The stem cells derived from embryonic cells [13], adipocytes [10], and urine [19] cannot express desmin at both gene and protein levels before differentiation. Interestingly, the expression of desmin demonstrated low basal level in noninduced DPSCs, which indicates DPSCs may be more suitable to be induced into SMCs for bladder tissue regeneration.

Belonging to the TGF-*β* family, TGF-*β*1 is deemed as a multifunctional growth factor, which regulates a wide range of biological processes, including cell proliferation, cell survival, and cell differentiation as well as cell migration [35, 36]. Studies have demonstrated that TGF-*β*1 plays a pivotal role in multilineage differentiation of mesenchymal stem cell. It promotes chondrogenic and osteogenic differentiation of BMMSCs [37–39], while it inhibits adipogenic differentiation of BMMSCs [39]. The effect of TGF-*β*1 for MSC differentiation involves all three lineages, which contributes to the development of SMCs from both embryonic stem cells-derived mesenchymal cells (ES-MCs) [13] and BMMSCs [15]. Gong and Niklason [40] also showed that TGF-*β*1 could inhibit MSC proliferation but increased the expression of calponin in a dose-dependent manner, which indicate that TGF-*β*1 not only initiates SMCs differentiation but also promotes further differentiation. TGF-*β*1-mediated SMCs differentiation was demonstrated through multiple signaling pathways, including Smad, p38 MAPK, and PI3K signaling [41]. In in vitro model system for vascular smooth muscle differentiation from human ES-MCs, TGF-*β*1 induced expression of SMCs markers depending on Smad [41], Jagged1-Notch [42], and PI3K signaling [41], as well as serum response factor (SRF)/CArG/myocardin [41], because either downregulation of Smad2, Smad3, and SRF or use of signaling inhibitors of Notch or PI3K blocks the expression of SMC markers. Additionally, both ERK/MAPK [43, 44] and Jagged1-Notch signaling [45] are involved in regulating the differentiation of BMMSCs into vascular SMCs. However, most of previous studies investigating the potential signaling pathways are mainly focused on the differentiation of vascular SMCs from ES-MCs and BMMSCs, and the control mechanism of the bladder SMCs differentiation from human DPSCs remains unknown. The PI's lab has recently discovered that Wnt-mediated GSK3*β*/*β*-catenin signaling was required in the process of human DPSCs towards human bladder SMCs (unpublished data). Therefore, further in-depth investigation shall be performed to fully evaluate the control mechanism of Wnt pathway during bladder SMCs differentiation, as well as the crosstalk between multiple pathways regulated by TGF-*β*1.

Smooth muscle is an involuntary nonstriated muscle which can be found in most parts of human organs. Among them, one of most important smooth muscles within the walls of blood vessels is specifically called vascular smooth muscle, which is mainly in the tunica media layer of the arterioles and veins. Apart from that, it also can be found in lymphatic vessels, the urinary bladder, uterus (termed uterine smooth muscle), male and female reproductive tracts, gastrointestinal tract, respiratory tract, arrector pili of skin, the ciliary muscle, and iris of the eye. Although SMCs within different organs might have similar morphological structure, their functions can be distinctively different depending on the hosting organs in which the SMCs are located. For example, SMCs in the stomach and intestines are responsible for propelling food through the digestive tract; SMCs in the vessels are responsible for the maintenance of normal blood pressure, whilst SMCs in the urinary bladder are responsible for storing urine and contraction for voiding. Therefore, in order to perform individual functions in different organs, SMCs tend to differentiate in an organ specific manner, determined by the individual niche accommodating the cells. Previous study has reported that BMMSCs can be induced to differentiate into vascular SMCs for vascular remodeling and repair after vascular injury, by conditioning them with vascular SMCs. Additionally, MSCs derived from bone marrow [46], dental pulp [47], and adipose tissue [48] have been regarded as promising candidates in the cell based therapies for muscle regeneration in muscular dystrophy patients, by coculturing them with skeletal myoblasts. The DPSCs/bladder SMCs coculturing protocol proposed in this study therefore can be a useful approach to differentiate toward bladder specific SMCs with potential urological functions. In our study, both TGF-*β*1 and conditioned medium derived from bladder SMCs are required for bladder SMCs differentiation. Previous study showed that several cytokines/growth factors including PDGF-BB, TGF-*β*1, HGF, and VEGF were detected in the SMCs-CM [15]. HGF/VEGF were expressed in high levels at the later stage of differentiation [15]. Further clarification is desired to fully reveal the synergistic and interactive functions of these growth factors in the regulation of bladder specific SMCs differentiation.

## 5. Conclusion

This study demonstrates that DPSCs can be induced to differentiate into bladder associated SMCs when treated with a combination of CM from bladder SMCs and TGF-*β*1. The multipotency and high proliferation of A32 along with its ability to differentiate into bladder SMCs are demonstrated for the first time. More work is required to investigate the related signaling pathways controlling the bladder SMCs differentiation and to evaluate the function of DPSCs differentiated bladder SMC-like cells after transplantation* in vivo*. This study represents a step forward in providing a promising alternative source of cells that are obtained by least invasive procedure without ethical issues, for future research in urinary bladder tissue engineering.

## Figures and Tables

**Table 1:** 

Genes	Forward and reverse primers	Accession number
GAPDH	5′-GCACCGTCAAGGCTGAGAAC-3′	NM_002046.3
	5′-TGGTGAAGACGCCAGTGGA-3′	

CD105	5′-GAAACAGTCCATTGTGACCTTCAG-3′	NM_001114753.2
	5′-GATGGCAGCTCTGTGGTGTTGACC-3′	

CD73	5′-GTCGCGAACTTGCGCCTGGCCGCCAAG-3′	NM_001204813.1
	5′-TGCAGCGGCTGGCGTTGACGCACTTGC-3′	

CD44	5′-CATCTACCCCAGCAACCCTA-3′	NM_000610.3
	5′-CTGTCTGTGCTGTCGGTGAT-3′	

CD90	5′-ATGAACCTGGCCATCAGCATCG-3′	NM_006288.3
	5′-CACGAGGTGTTCTGAGCCAGCA-3′	

CD34	5′-ACAGGAGAAAGGCTGGGCGAAGACCCT-3′	NM_001025109.1
	5′-TCCCCTGGGGGTTCCTGTATTGCGGCA-3′	

*α*-SMA (ACTA2)	5′-CCGGTTGGCCTTGGGGTTCAGGGGTGCC-3′	NM_001141945.1
	5′-TCTCTCCAACCGGGGTCCCCCCTCCAGCG-3′	

Myosin (MYH11)	5′-AAGAAAGACACAAGTATCACGGGAGAGC-3′	NM_001040113.1
	5′-TGTCACATTAATTCCCATGAGGTGGCAA-3′	

Desmin	5′-CACCATGAGCCAGGCCTACTCGTCCA-3′	NM_001927.3
	5′-GGCAGCCAAATTGTTCTCTGCTTCTTCC-3′	

Calponin	5′-GGCTCCGTGAAGAAGATCAATGAGTCAA-3′	NM_001299.4
	5′-CCCTAGGCGGAATTGTAGTAGTTGTGTG-3′	
